# Supplementary material for: Wearable Sleep Measures May Improve Machine Learning Prediction of Home-Based Pulmonary Rehabilitation Engagement Among Patients With Chronic Obstructive Pulmonary Disease: A Proof-of-Concept Study
Source: Mayo Clin Proc Digit Health. 2026 Mar 6;4(2):100345. doi: 10.1016/j.mcpdig.2026.100345 (PMC13053982; doi:10.1016/j.mcpdig.2026.100345)
Supplement: Supplementary Material [file mmc1.pdf]

Supplementary Material

Supplementary Table 1

SENSITIVITY ANALYSIS #1 (Composite Sleep Health Score only with no other predictors)

	Sensitivity (%)	Specificity (%) (95% CI)	Accuracy (%) (95% CI)	AUC (95% CI)	p-value for AUCs
Logistic regression model					0.12
Without Composite Sleep Health Score	85.0 (75.3-92.0)	31.8 (18.6-47.6)	66.1 (57.1-74.4)	0.708 (0.611-0.805)	
With Composite Sleep Health Score	91.2 (82.8-96.4)	25.0 (13.2-40.3)	67.7 (58.8-75.9)	0.595 (0.484-0.706)	
Decision tree model					0.92
Without Composite Sleep Health Score	87.5 (78.2-93.8)	36.4 (22.4-52.2)	69.4 (60.4-77.3)	0.511 (0.403-0.619)	
With Composite Sleep Health Score	88.8 (79.7-94.7)	22.7 (11.5-37.8)	65.3 (56.3-73.6)	0.520 (0.405-0.635)	
Support vector machine					0.65
Without Composite Sleep Health Score	96.2 (89.4-99.2)	18.2 (9.2-32.7)	68.5 (59.6-76.6)	0.593 (0.485-0.701)	
With Composite Sleep Health Score	93.8 (86.0-97.9)	13.6 (5.2-27.4)	65.3 (56.3-73.6)	0.559 (0.450-0.669)	
Naïve Bayes classifier					0.09
Without Composite Sleep Health Score	97.5 (91.3-99.7)	22.7 (11.5-37.8)	71.0 (62.1-78.8)	0.680 (0.611-0.805)	
With Composite Sleep Health Score	88.8 (79.7-94.7)	22.7 (11.5-37.8)	65.3 (56.3-73.6)	0.548 (0.484-0.706)	

Supplementary Table 2

SENSITIVITY ANALYSIS #2 (No Composite Sleep Health Score, replaced with sleep regularity direct measures)

	Sensitivity (%)	Specificity (%) (95% CI)	Accuracy (%) (95% CI)	AUC (95% CI)	p-value for AUCs
Logistic regression model					0.31
Without Composite Sleep Health Score	87.5 (78.2-93.8)	31.8 (18.6-47.6)	67.7 (58.8-75.9)	0.695 (0.599-0.791)	
With Composite Sleep Health Score	86.2 (76.7-92.9)	27.3 (15.0-42.8)	65.3 (56.3-73.6)	0.627 (0.517-0.737)	
Decision tree model					0.74
Without Composite Sleep Health Score	73.8 (62.7-83.0)	40.9 (26.3-56.8)	62.1 (52.9-70.7)	0.533 (0.424-0.641)	
With Composite Sleep Health Score	71.2 (60.0-80.8)	43.2 (28.3-59.0)	61.3 (52.1-69.9)	0.560 (0.451-0.669)	
Support vector machine					0.68
Without Composite Sleep Health Score	93.8 (86.0-97.9)	22.7 (11.5-37.8)	68.5 (59.6-76.6)	0.566 (0.456-0.675)	
With Composite Sleep Health Score	92.5 (84.4-97.2)	18.2 (8.2-32.7)	66.1 (57.1-74.4)	0.595 (0.489-0.702)	
Naïve Bayes classifier					0.08
Without Composite Sleep Health Score	95.0 (87.7-98.6)	18.2 (8.2-32.7)	67.6 (58.8-75.9)	0.679 (0.599-0.791)	
With Composite Sleep Health Score	82.5 (72.4-90.1)	22.7 (11.5-37.8)	61.3 (52.1-69.9)	0.554 (0.517-0.737)	
